# Varenicline as a Repurposable Drug Candidate for ADRD Prevention

**DOI:** 10.64898/2026.09.17.26363232

**Published:** 2026-09-20

**Authors:** Phillip Ma, Jiaxu Zhou, Yan Cheng, John E. McGeary, Tracey H Taveira, Ali Ahmed, Wen-Chih Wu, Edward Zamrini, Brittany N. Dugger, Louise Nicole C. Sevilla, Joel Anthony Nations, Yijun Shao, Ying Yin, Debby W. Tsuang, Mark W Logue, Siamack Ayandeh, Charles Faselis, Stuart J. Nelson, Qing Zeng-Treitler

## Abstract

Objective: Effective strategies to prevent Alzheimer's disease and related dementias (ADRD) are limited. Implementing our Medication-Wide Association Study (MWAS+), we highlighted varenicline as a candidate. This study evaluated the association between varenicline use and risk of ADRD relative to nicotine replacement therapy (NRT). Methods: Using the All of Us Research Database, we identified adults aged 50 years or older with available lifestyle survey responses regarding tobacco use history who initiated smoking cessation therapy with either varenicline or NRT between 2006 and 2023. We limited our cohort to incident monotherapy users and designated the index date as the first prescription. Individuals with baseline ADRD or no follow-up were excluded. Baseline characteristics included demographics, comorbidities, BMI, and prior medication exposures. Outcomes were incident ADRD and a composite outcome of ADRD or death. Findings: The cohort consisted of 9,584 individuals (1,801 varenicline users and 7,783 NRT users). After propensity score matching, there were 1,758 individuals in each group with balanced baseline characteristics. Varenicline use was associated with a reduced risk of ADRD (HR 0.516, 95% CI: 0.353-0.754) and the composite outcome ADRD or death (HR 0.657, 95% CI: 0.501-0.861). Kaplan-Meier curves also demonstrated varenicline's sustained benefits through the study period. Implications: Varenicline use is associated with a lower risk of ADRD among older adults receiving smoking cessation therapy. Further investigation of varenicline as a potential strategy for ADRD prevention is warranted. These findings underscored the value of explainable AI-guided approaches to drug repurposing.

## Full Text Availability

The license terms selected by the author(s) for this preprint version do not permit archiving in PMC. The full text is available from the preprint server.

